# The Prevalence of Mental Disorders Among Children and Adolescents in the Child Welfare System: A Systematic Review and Meta-Analysis

**DOI:** 10.1097/MD.0000000000002622

**Published:** 2016-02-18

**Authors:** Guillaume Bronsard, Marine Alessandrini, Guillaume Fond, Anderson Loundou, Pascal Auquier, Sylvie Tordjman, Laurent Boyer

**Affiliations:** From the Maison Départementale de l’Adolescent et CMPPD (GB), Conseil Départemental des Bouches-du-Rhône, Marseille, France; Service d’épidémiologie et d’économie de la santé (MA, PA, LB), Pôle de Santé Publique, Hôpital La Timone, Assistance Publique—Hôpitaux de Marseille, Marseille, France; Aix-Marseille University (MA, AL, PA, LB), Marseille, France; INSERM U955 (GF), Translational Psychiatry team, Paris Est University, DHU Pe-PSY, Pôle de Psychiatrie des Hôpitaux Universitaires H Mondor, Créteil, France; Fondation Fondamental (GF), Fondation de Coopération en Santé Mentale; Network of Expert Centres for Schizophrenia (GF), Créteil, France; Laboratoire Psychologie de la Perception (ST), Université Paris Descartes, Paris, France; and Service Hospitalo-Universitaire de Psychiatrie de l’Enfant et de l’Adolescent de Rennes (ST), Université de Rennes 1, Rennes, France.

## Abstract

It remains unclear whether children and adolescents in the child welfare system (CWS) exhibit a higher prevalence of mental disorders compared with the general population. The objective of this study was to perform a systematic review and meta-analysis to assess the prevalence of mental disorders in the CWS.

All of the epidemiological surveys assessing the prevalence of mental disorders in children and adolescents in the CWS were included. The pooled prevalence was estimated with random effect models. Potential sources of heterogeneity were explored using meta-regression analyses.

Eight studies provided prevalence estimates that were obtained from 3104 children and adolescents. Nearly 1 child or adolescent of every 2 (49%; 95% confidence interval (CI) 43–54) was identified as meeting criteria for a current mental disorder. The most common mental disorder was disruptive disorder (27%; 95% CI 20–34), including conduct disorder (20%; 95% CI 13–27) and oppositional defiant disorder (12%; 95% CI 10–14). The prevalence of attention-deficit/hyperactivity disorder was estimated to be 11% (95% CI 6–15). The prevalence estimates of anxiety and depressive disorders were 18% (95% CI 12–24) and 11% (95% CI 7–15). Posttraumatic stress disorder had the lowest prevalence (4%; 95% CI 2–6).

High prevalences of mental disorders in the CWS were reported, which highlights the need for the provision of qualified service. The substantial heterogeneity of our findings is indicative of the need for accurate epidemiological data to effectively guide public policy.

## Abstract 2

Supplemental Digital Content is available in the text

**Key Messages Box**

Mental disorders affect a substantially greater proportion of children and adolescents in the child welfare system than in the general population. The 49% pooled prevalence for any mental disorder is nearly 4-fold greater than the prevalence among the general population.
The relatively low number of psychiatric epidemiological surveys and the substantial heterogeneity of our findings are indicative of the need for accurate epidemiological data to inform and guide effective public policy.
Given the importance of mental disorders in this population, the poor prognoses of the complex mental states and the high cost to society, it is unfortunate that this population of youths suffering from mental disorders in the child welfare system does not benefit from greater attention.

## INTRODUCTION

The literature on the prevalence of mental disorders among children and adolescents in the general population has significantly increased over the last years.^1–3^ Compared with the general population, little is known about the prevalence of mental disorders among children and adolescents in the child welfare system (CWS)^4^ specifically because they are often excluded from epidemiological studies because of their high mobility and difficulties surrounding parental responsibility and informed consent.^5,6^ However, this issue is far from uncommon in Western countries, in which the rate of the placement of children and adolescents outside the home has been estimated to be approximately 5 per 1000, and the overall rate of children and adolescents in the CWS has been estimated to be 18 per 1000.^7,8^ This population has constantly been increasing for over the last 20 years.^9^ The literature focusing on this issue highlights that the children and adolescents involved in the CWS are very vulnerable in terms of psychological disturbances due to histories of child abuse and neglect, separation from their biological parents, or placement instability.^10–12^ Several studies have reported that the prevalence of mental disorders among this population is greater than in the general population, but these findings are highly heterogeneous (eg, the prevalence estimates of anxiety disorders range from 4% to 32%.^4,5,9–11,13–15^ In addition to their important health care needs, these children and adolescents experience barriers in accessing appropriate and continuous care that worsen their prognoses.^15–19^ These children and adolescents, particularly maltreated children who are placed in out-of-home care, are more likely to be involved in the juvenile justice system and to experience recidivism.^20^ All of these factors result in substantial economic effects and major costs to society.^21,22^

More reliable estimates of the prevalence of mental disorders and the identification of the sources of heterogeneity among children and adolescents in the CWS are needed to inform public policy and to develop adapted psychiatric services, training for professionals, and research planning.^2^ The most recent review dates from 2008 but was descriptive and did not attempt a quantitative synthesis of the evidence or to explore the heterogeneity between studies.^15^ The objective of the present study was to assess the prevalence of mental disorders in children and adolescents in the CWS in a first systematic review and meta-analysis.

## METHODS

### Study Selection

All of the epidemiological studies assessing the prevalence of mental disorders in children and adolescents in the CWS were included in the present work. To identify the relevant studies, we reviewed the following databases up to January 30, 2015, and the beginning years of searching were selected according to the creation date of each database: PubMed (from 1966), ERIC (from 1964), FRANCIS (from 1972), PsycARTICLES (from 1894), PsychINFO (from 1806), and Science Direct (from 2006). A specific search strategy was developed based on a combination of the following terms: (mental disorders OR psychiatric disorders) AND (epidemiology OR prevalence OR survey) AND (child OR adolescent OR youth) AND (child welfare OR foster OR residential OR out of home OR local authority care OR child maltreatment OR youth welfare institution). Two persons on the reviewing team (GB and MA) independently reviewed the references and abstracts retrieved by the search and assessed the completeness of the data abstraction. We used a structured data abstraction form to ensure the consistency of the appraisals of each study. The investigators were contacted and asked to provide data to supplement incomplete reports in the original articles when necessary.

### Criteria for Selecting the Articles

Studies were included if they met the following criteria:

1. Design: epidemiological studies;
2. Participants: children and adolescents involved in the child welfare system, that is, those placed in foster care homes, residential group homes, or others (e.g., independent living placements and those living with their parents part of the time);
3. Psychiatric diagnoses were based on standardized diagnostic criteria using an international classification of diseases, that is, the Classification of Mental and Behavioral Disorders (ICD-8, ICD-9, and ICD-10) or the Diagnostic and Statistical Manual of Mental Disorders (DSM-III, DSM-III-R, DSM-IV, and DSM-V), based on structured or semistructured interviews.
4. The prevalence rates were reported for current psychiatric disorders.

There were no language or date restrictions. Studies that reported only lifetime (and not current) diagnoses were excluded from the analyses.

### Selection of Studies and Data Extraction

Two authors (GB and MA) screened the titles and abstracts of the database records and retrieved the full texts for eligibility assessment and independently examined the full-text records for eligibility. Disagreements were resolved by consensus discussion.

The articles of the studies were then independently reviewed by 2 of the authors (GB and MA). The data were independently extracted into a standard electronic form that included the following: the name of the first author, date of publication, country, representativeness of the sample, type of placement, type of population, sex ratio, mean age, age range, sample size, study design (ie, a 1-step procedure that involved applying the diagnostic interview to the whole sample or a 2-step procedure that applied screening instruments followed by diagnostic interviews for only those participants who screened positively), participation rate of the screening sample, screening instrument used in the studies with 2-step procedures, participation rate of the diagnostic sample, diagnostic instrument, type of diagnostic instrument (ie, structured or semistructured), diagnostic criteria, informants (ie, youths, parents or caregivers, and teachers), and functional impairment (requirement for the diagnosis and definition). Any discrepancies were resolved by consensus with a third reviewer (LB).

### Assessing the Methodological Qualities of Included Studies

The methodological qualities of the included studies were assessed independently by 2 of the authors (MA and LB) using a validated checklist of items for observational studies in epidemiology.^23^ Any discrepancies were resolved by consensus with a third reviewer (PA).

### Statistical Analyses

The overall pooled-prevalence was estimated with a random effects model^24^ that accounted for between-study heterogeneity by weighting the studies similarly. Heterogeneity was assessed using the I^2^ statistic, which represents the percentage of variance that is due to between-study factors rather than sampling error.^25,26^ We considered I^2^ values >50% as indicative of large heterogeneity. We used funnel plots and the Egger regression intercept (which assesses the degree of funnel plot asymmetry based on the intercept from the regression of the standard normal deviates against the precision) to estimate the risk of bias.^27^ Forest plots were generated to demonstrate the prevalence with the corresponding confidence intervals (CIs) for each study and the overall random effects pooled estimate. The potential sources of heterogeneity were investigated by arranging groups of studies according to potentially relevant characteristics into subgroups and meta-regression analyses. The factors that were individually examined included the following: date of publication (in years), country (2 groups: United-States of America and Europe), sex ratio, mean age, study design (2 groups: 1-step procedure and 2-step procedure), sample size (2 groups: n < 300 and n ≥ 300), type of diagnostic instrument (2 groups: structured and semistructured instruments), diagnostic criteria (2 groups: DSM-III-R and DSM-IV/ICD-10), participation rate (2 groups: ≥70% and <70%), informants (2 groups: youths, parents or caregivers and teachers and only youths), and functional impairment (2 groups: required for diagnosis and not required). The factors associated with heterogeneity at *P* < 0.05 were subsequently included in multivariate meta-regression models.

The analyses were performed with Comprehensive Meta-Analysis Software (version 2.0, National Institute of Health) and the STATA statistical software package, version 10 (StataCorp 2007, College Station, TX) using the command metareg (for meta-regression).

### Role of the Funding Source

No drug manufacturing company was involved in the study design, data collection, data analysis, data interpretation, writing of the report, or in the decision to submit the report for publication. All authors saw and approved the final version of the article. The corresponding author had full access to all data and decided to submit for publication.

## RESULTS

### Literature Search

**FIGURE 1:**
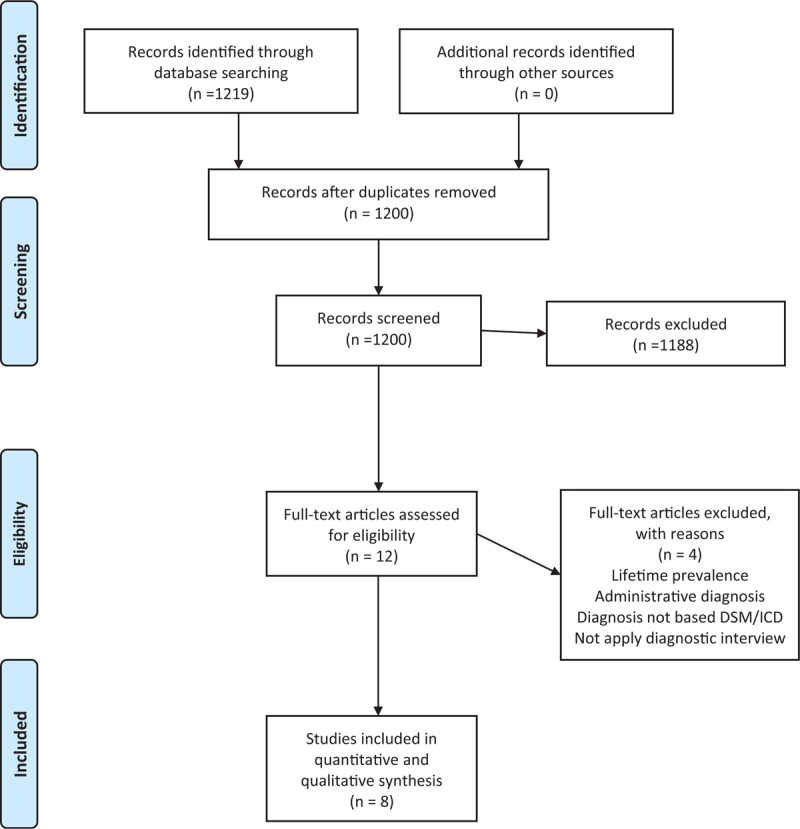
PRISMA 2009 flow diagram of review process and study selection.

The PRISMA statement flowchart (Figure 1) describes the literature screening, study selection, and reasons for exclusion. A total of 8 studies met the inclusion criteria for the present investigation^4,5,9–11,13–15^ and were ultimately included in the meta-analysis.

### Included Studies: Main Characteristics

The methodological qualities of the included studies are presented in Table 1, the characteristics of studies are presented in Tables 2 and 3, and the characteristics of the 7 diagnostic instruments (ie, the K-SADS-PL, K-SADS-P, DISC-IV/C-DISC-IV, DISC-2.25, DIS-IV, DAWBA, and DISYPS-KJ) are presented in Table 4.^28–35^ The 8 studies were published from 1996^11^ to 2013^9^ and were performed in the following 5 different Western countries: 1 study was performed in France,^10^ 1 in Germany,^15^ 1 in Norway,^9^ 3 in the United Kingdom,^5,11,13^ and 2 in the United States.^4,14^ The sample sizes ranged from 48^13^ to 1253 subjects,^5^ and a total of 3104 children and adolescents were included. Regarding the type of placement, 2 studies included subjects from residential group homes,^10,15^ 1 study included subjects from foster care homes,^9^ 2 studies included subjects from both residential group and foster care homes,^11,13^ and 3 studies included subjects from any type of child welfare placement.^4,5,14^

**TABLE 1:**
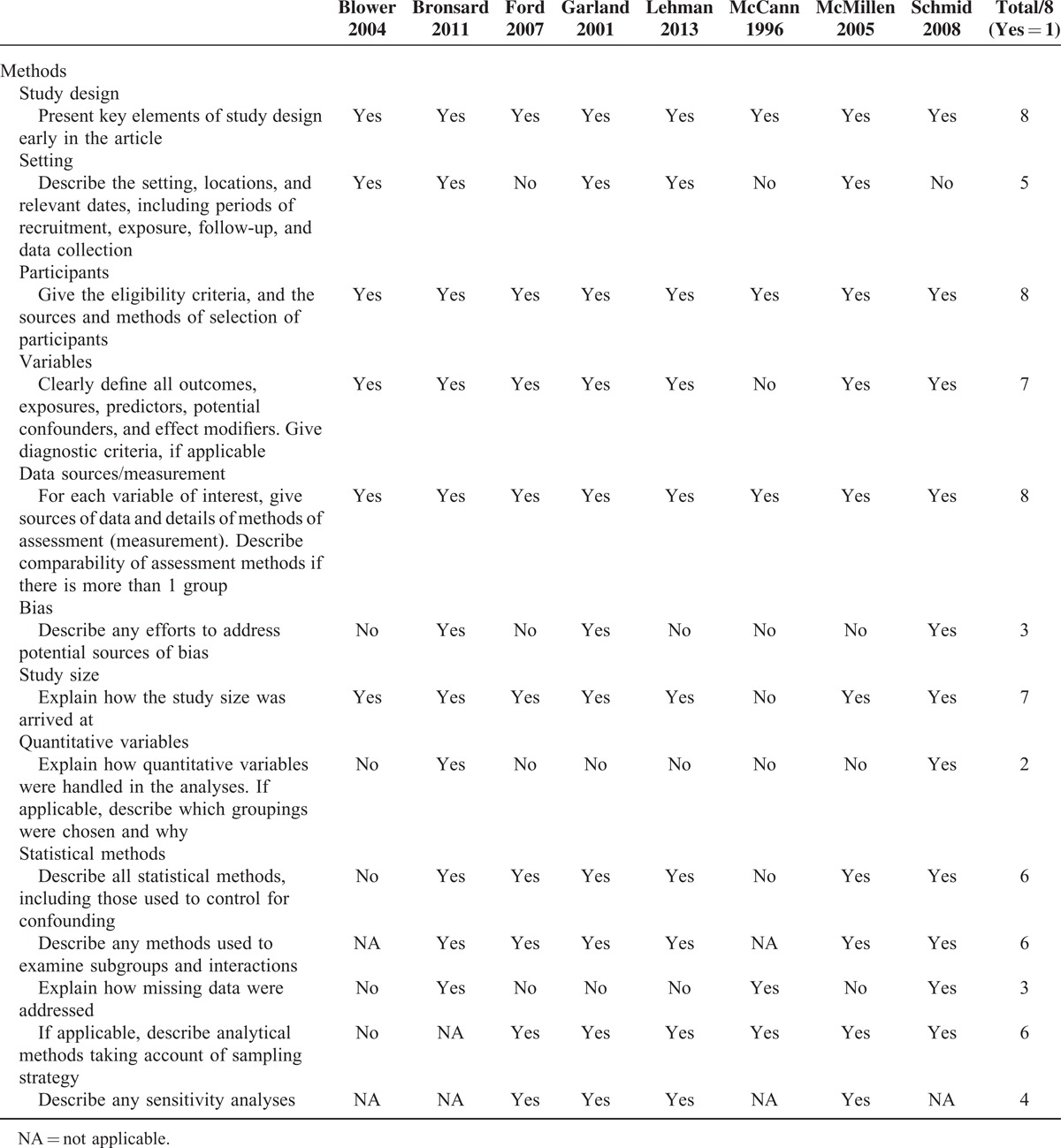
Methodological qualities of the included studies (n = 8)

**TABLE 2:**
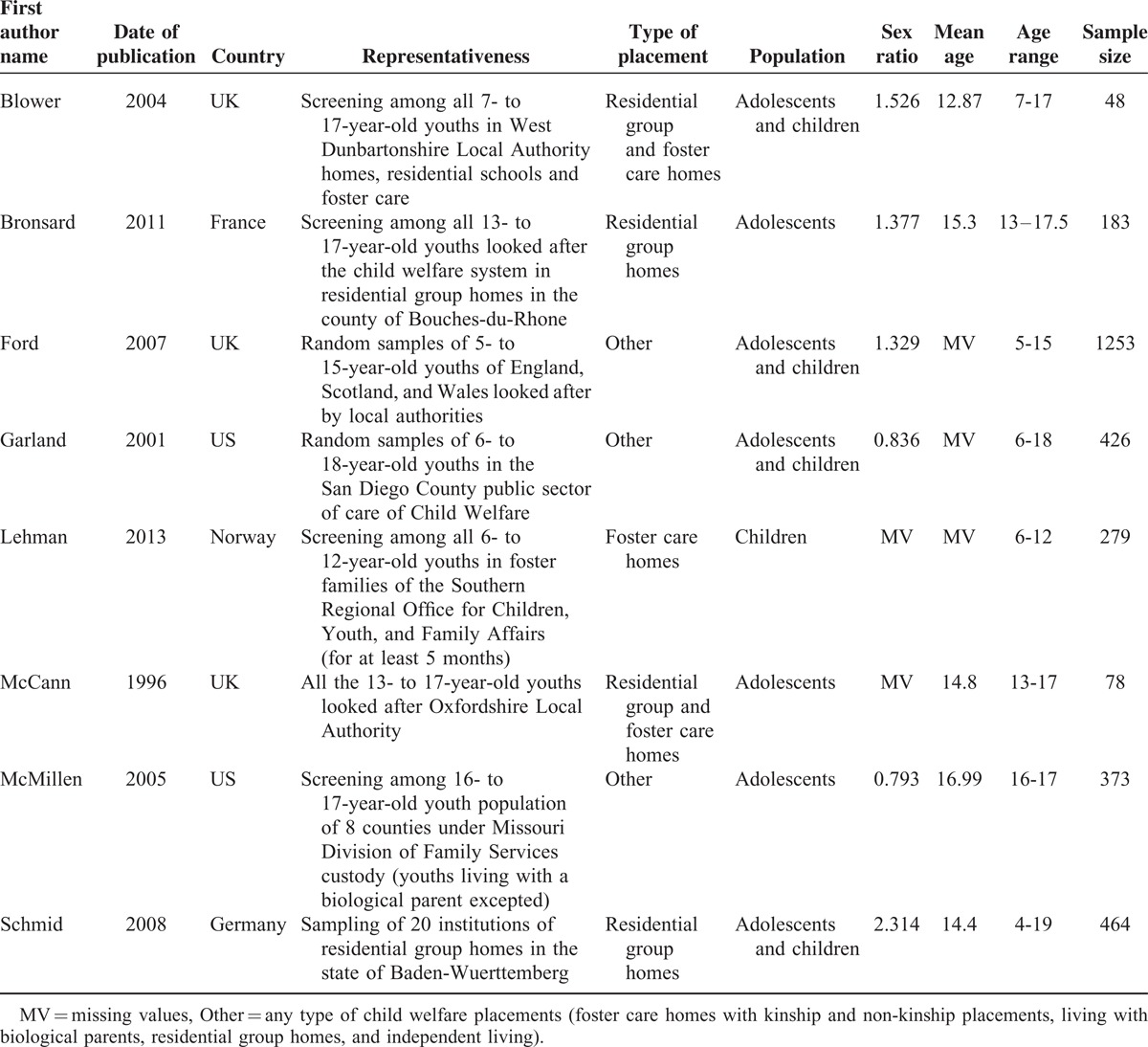
Characteristics of the included studies

**TABLE 3:**
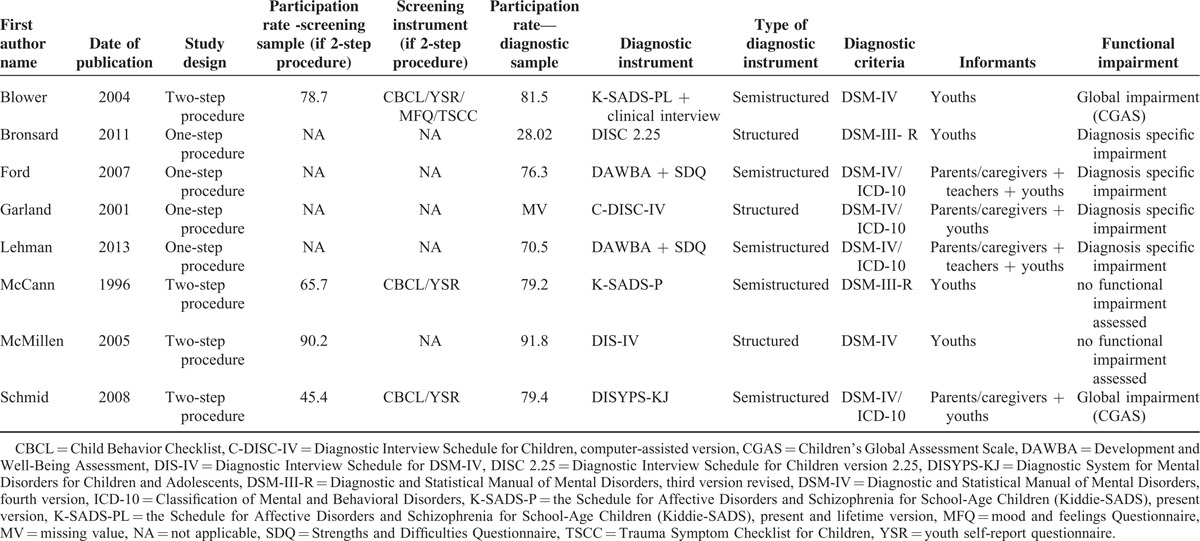
Characteristics of the included studies

**TABLE 4:**
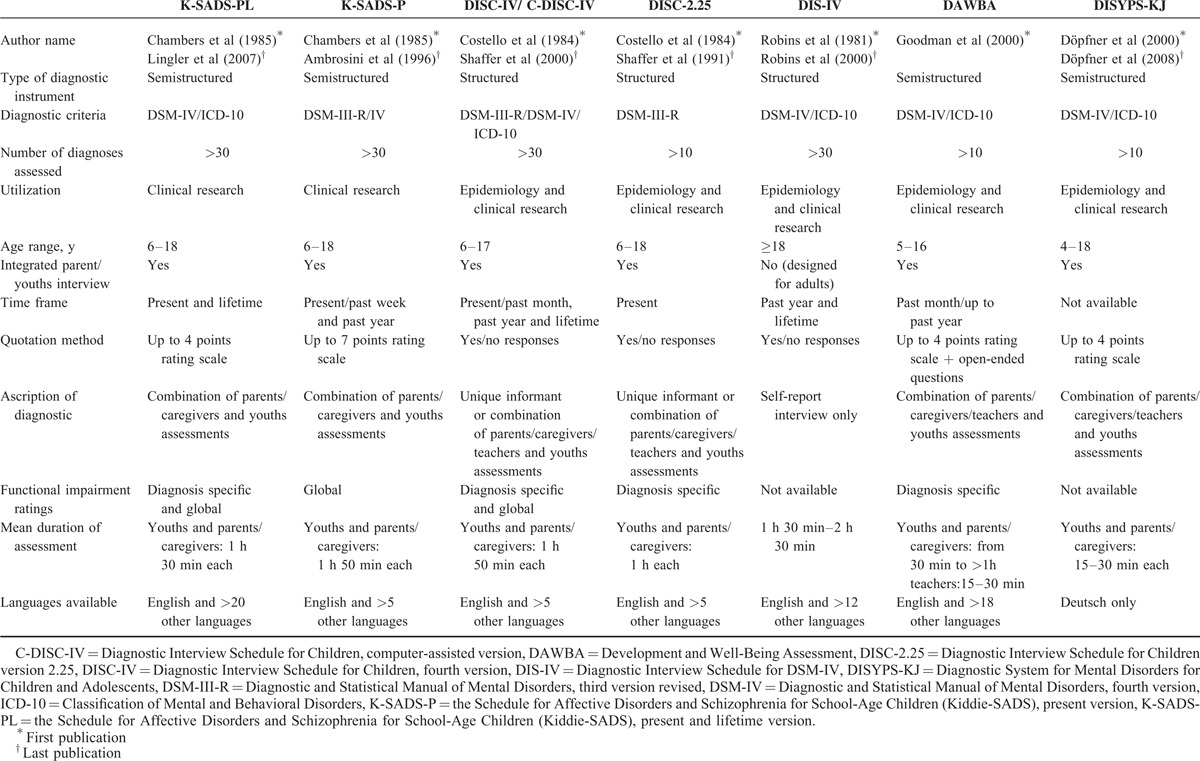
Characteristics of diagnostic instrument

### Prevalence Estimates

Our meta-analysis estimated the pooled prevalence rates of the individual diagnostic groups that were commonly reported by the studies. The funnel plots were rather asymmetrical for all of the mental disorders, which suggested potential publication bias (Appendix). However, the *P* value of the Egger regression intercept was >0.05, and the asymmetry was thus statistically nonsignificant.

#### Any Depressive Disorder

**FIGURE 2:**
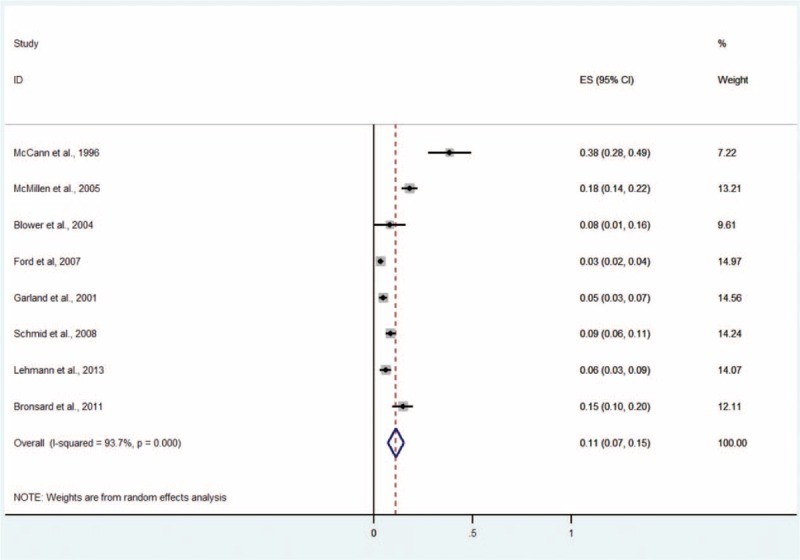
Prevalence of any depressive disorder in children and adolescents in the CWS. CWS = child welfare system.

Eight studies including 3104 subjects provided data about any depressive disorder, including major depressive disorder, bipolar depressive disorder, dysthymia, and other and minor depressive disorders not otherwise specified (NOS) (Figure 2).^4,5,9–11,13–15^ The prevalence estimates ranged from 3% to 38%. The random effects pooled prevalence estimate was 11% (95% CI 7–15, *P* < 0.001, I^2^ = 93.7%).

#### Major Depressive Disorder

**FIGURE 3:**
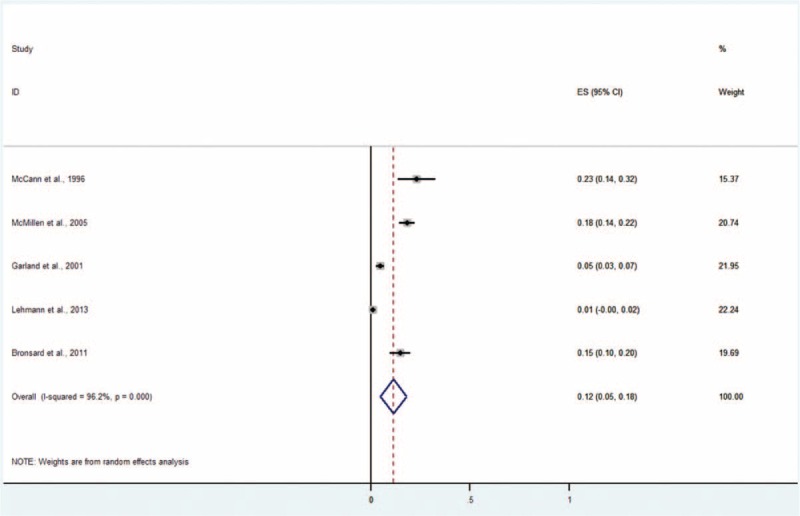
Prevalence of major depressive disorder in children and adolescents in the CWS. CWS = child welfare system.

The major depressive disorder was analyzed subsequently because this subtype of depressive disorder is one of the most important challenges in global mental health,^36^ especially in youths.^37^ Five studies including 1339 subjects provided data about major depressive disorder (Figure 3).^4,9–11,14^ The prevalence estimates ranged from 1% to 23%. The random effects pooled prevalence estimate was 12% (95% CI 5–18, *P* < 0.001, I^2^ = 96.2%).

#### Any Disruptive Disorder

**FIGURE 4:**
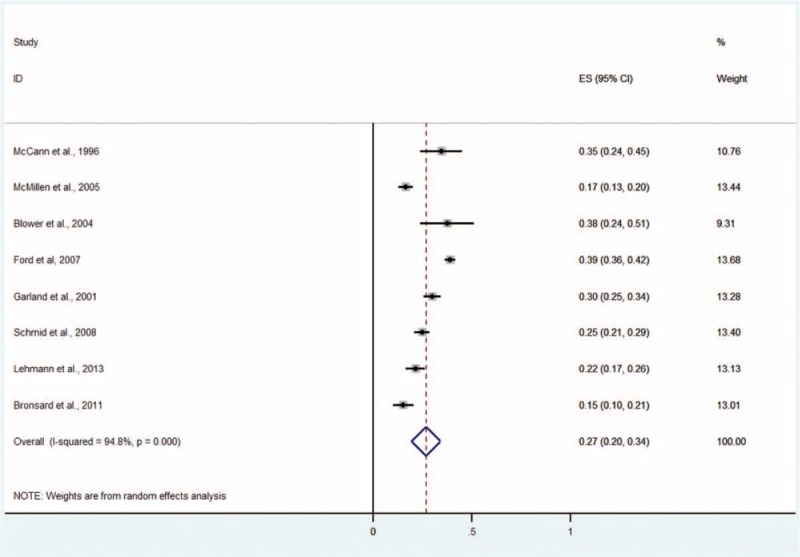
Prevalence of any disruptive disorder in children and adolescents in the CWS. CWS = child welfare system.

Eight studies including 3104 subjects provided data about any disruptive disorder including conduct disorder, oppositional defiant disorder (ODD), and other disruptive disorders (Figure 4).^4,5,9–11,13–15^ The prevalence estimates ranged from 15% to 39%. The random effects pooled prevalence estimate was 27% (95% CI 20–34, *P* < 0.001, I^2^ = 94.8%).

#### Conduct Disorder

**FIGURE 5:**
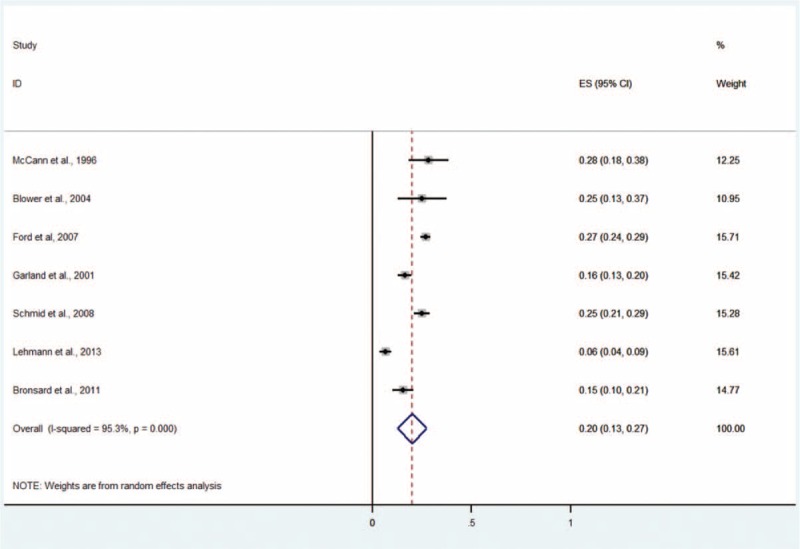
Prevalence of any conduct disorder in children and adolescents in the CWS. CWS = child welfare system.

Seven studies including 2731 subjects provided data about conduct disorder (Figure 5).^5,9–11,13–15^ The prevalence estimates ranged from 6% to 28%. The random effects pooled prevalence estimate was 20% (95% CI 13–27, *P* < 0.001, I^2^ = 95.3%).

#### Oppositional Defiant Disorder

**FIGURE 6:**
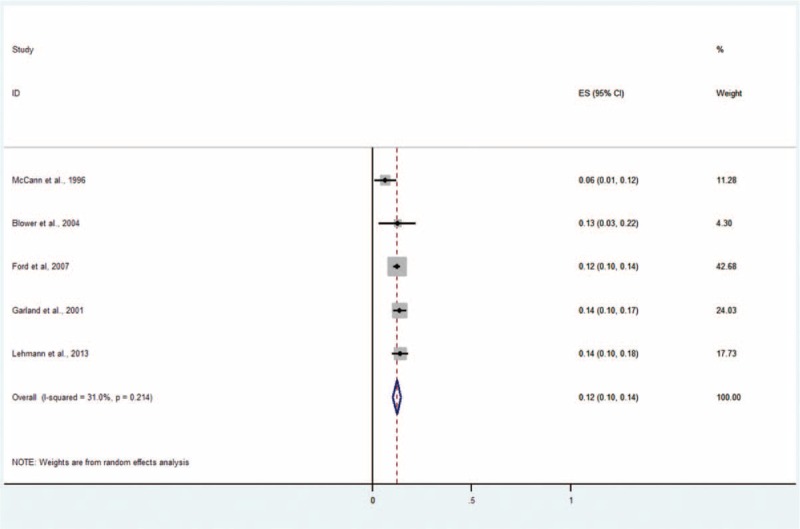
Prevalence of any ODD in children and adolescents in the CWS. CWS = child welfare system, ODD = oppositional defiant disorder.

Five studies including 2084 subjects provided data about ODD (Figure 6).^5,9,11,13,14^ The prevalence estimates ranged from 6% to 14%. The random effects pooled prevalence estimate was 12% (95% CI 10–14, *P* < 0.214, I^2^ = 31.0%).

#### Attention-Deficit/Hyperactivity Disorder

**FIGURE 7:**
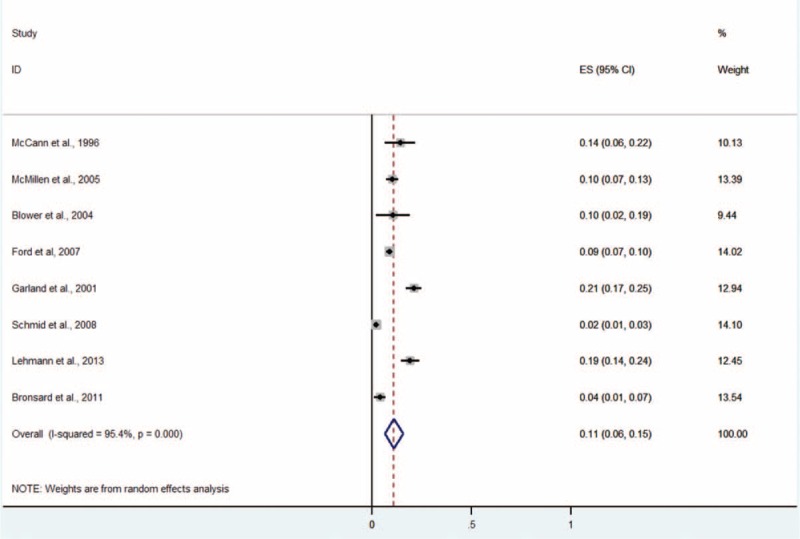
Prevalence of ADHD in Children and Adolescents in the CWS. CWS = child welfare system, ADHD = attention-deficit/hyperactivity disorder.

Eight studies including 3104 subjects provided data for attention-deficit/hyperactivity disorder (ADHD) (Figure 7).^4,5,9–11,13–15^ The prevalence estimates ranged from 2% to 21%. The random effects pooled prevalence estimate was 11% (95% CI 6–15, *P* < 0.001, I^2^ = 95.4%).

#### Any Anxiety Disorder

**FIGURE 8:**
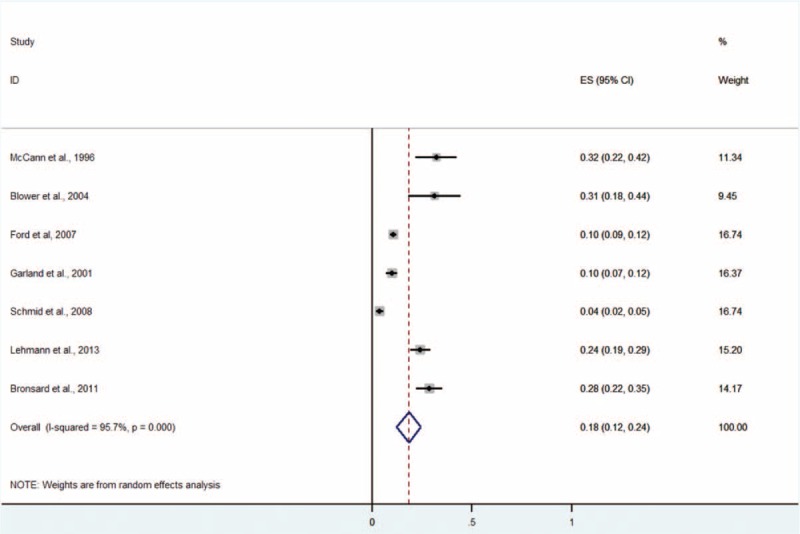
Prevalence of any anxiety disorder in children and adolescents in the CWS. CWS = child welfare system.

Seven studies including 2731 subjects provided data about any anxiety disorder, including generalized anxiety disorder, overanxious disorder, separation-anxiety disorder, specific phobia, social phobia, panic disorder, obsessive compulsive disorder, posttraumatic stress disorder, and other anxiety disorders NOS (Figure 8).^5,9–11,13–15^ The prevalence estimates ranged from 4% to 32%. The random effects pooled prevalence estimate was 18% (95% CI 12–24, *P* < 0.001, I^2^ = 95.7%).

#### Posttraumatic Stress Disorder

**FIGURE 9:**
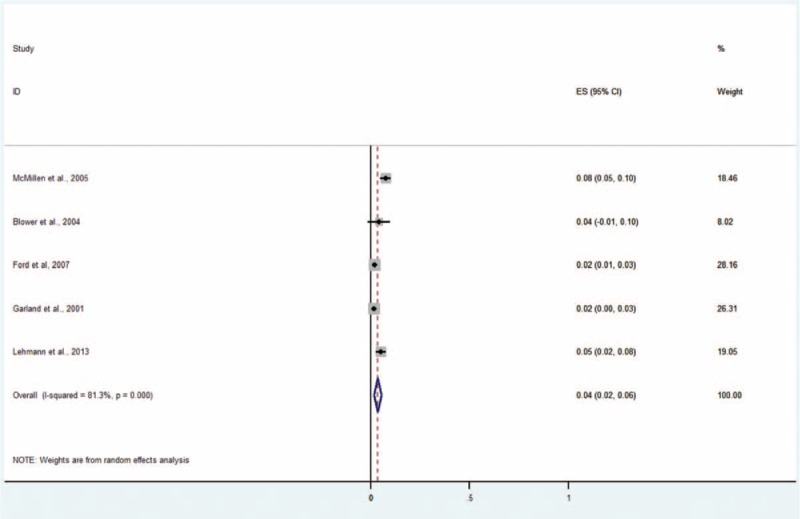
Prevalence of posttraumatic stress disorder in children and adolescents in the CWS. CWS = child welfare system.

Five studies including 2379 subjects provided data about posttraumatic stress disorder (Figure 9).^4,5,9,13,14^ The prevalence estimates ranged from 2% to 8%. The random effects pooled prevalence estimate was 4% (95% CI 2–6, *P* < 0.001, I^2^ = 81.3%).

#### Any Mental Disorder

**FIGURE 10:**
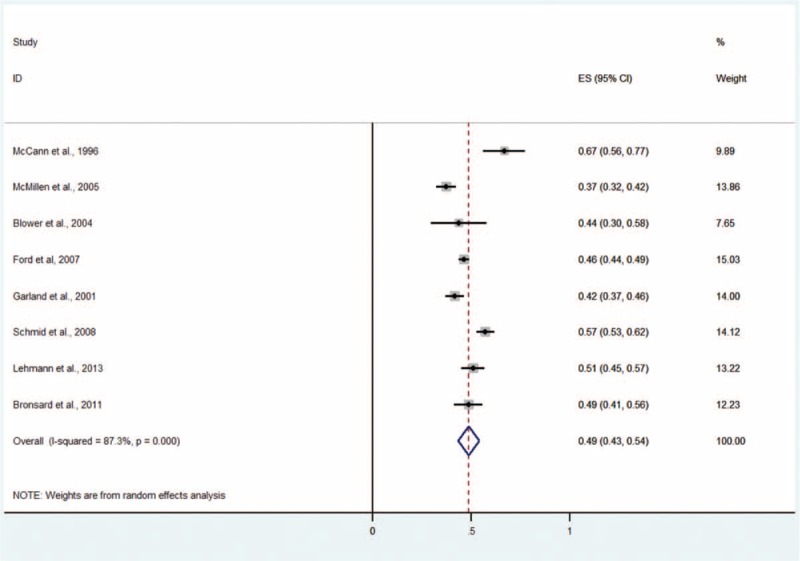
Prevalence of any mental disorder in children and adolescents in the CWS. CWS = child welfare system.

Eight studies including 3104 patients provided data about any mental disorder (Figure 10).^4,5,9–11,13–15^ The prevalence estimates ranged from 37% to 67%. The random effects pooled prevalence estimate was 49% (95% CI 43–54, *P* < 0.001, I^2^ = 87.3%).

### Meta-Regression Analyses

The results of the individual variable meta-regression models for each mental disorder are presented in Table 5. The final multivariate model identified diagnostic criteria and functional impairment (β = −0.12, se[β] = 0.04, *P* < 0.01; and β = −0.15, se[β] = 0.04, *P* < 0.001, respectively) as significant moderators of the prevalence estimate of any depressive disorder, informants (β = −0.12, se[β] = 0.04, *P* < 0.01) as a significant moderator of the prevalence estimates of major depressive disorder, functional impairment (β = 0.06, se[β] = 0.03, *P* < 0.05) as a significant moderator of the prevalence estimates of ODD, mean age (β = 0.03, se[β] = 0.01, *P* < 0.001) as a significant moderator of the prevalence estimates of ADHD, sample size (β = −0.17, se[β] = 0.05, *P* < 0.001) as a significant moderator of the prevalence estimates of any anxiety disorder, and sex ratio and country (β = 0.09, se[β] = 0.03, *P* < 0.001 and β = −0.09, se[β] = 0.04, *P* < 0.05, respectively) as significant moderators of the prevalence estimates of any mental disorders. No significant moderator was found for disruptive and conduct disorders.

**TABLE 5:**
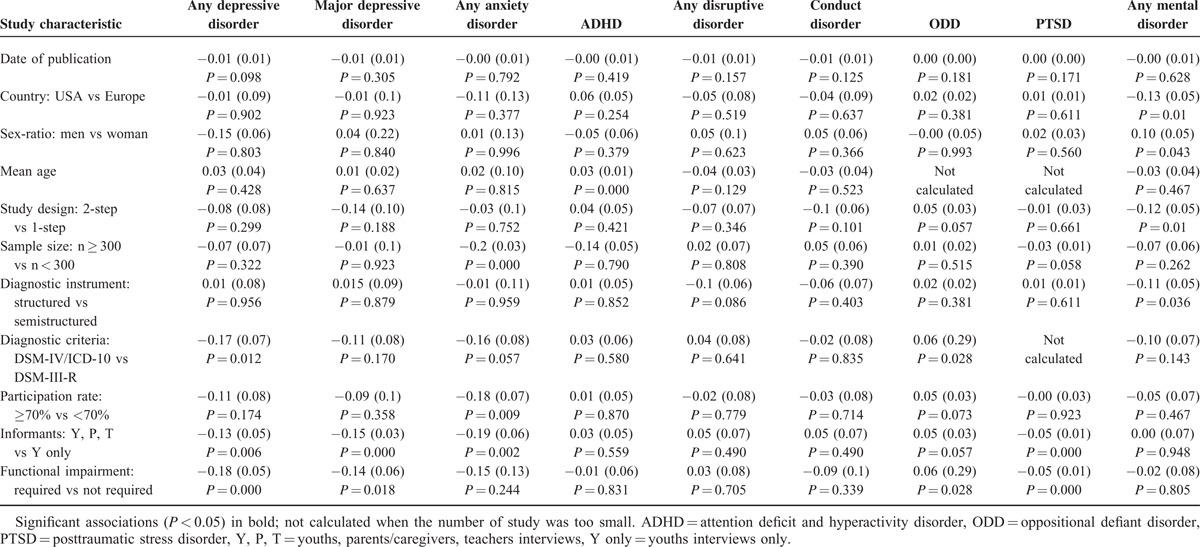
Results of individual variable meta-regression model for each diagnosis values of β, Se (β), and the significance of β for each study characteristic

## DISCUSSION

This systematic review of children and adolescents in the CWS identified 8 surveys that included 3104 subjects.^4,5,9–11,13–15^

Our findings suggest that mental disorders affect a substantially greater proportion of children and adolescents in the CWS that in the general population. The 49% pooled prevalence for any mental disorder estimated by our meta-analysis is nearly 4-fold greater than the 13.4% pooled prevalence among the general children and adolescent population.^2^ Bowlby attachment theory underscores the central role of child-to-parent attachment in a child's development and mental health and may explain the high prevalence observed in our work.^38–40^ Several empirical studies and review reported connections between attachment insecurities and vulnerability to mental disorders.^41,42^ In children and adolescents in the CWS, adverse experiences, such as maltreatment and serious neglect, contribute to reducing the likelihood of creating a secure attachment that is crucial for developmental health.^9,12^ In addition, although the CWS should provide safe alternative homes, multiple placements and temporary or disrupted relationships with caregivers can also potentially prevent the children from forming secure attachments.^43^

Our findings indicate that externalized disorders are the primary main problem in children and adolescents in the CWS. The most common mental disorder was disruptive disorder (27%). The prevalence of conduct disorder was 20%, and the prevalence for ODD was 12%, which are 10 and 3 times more frequent than the prevalences in the general population, respectively. Notably, ADHD was also approximately 3 times more frequent in the CWS children than in the general population.^2,44,45^ This high prevalence of externalized disorders may be explained by the fact that the symptoms of conduct disorder, such as property loss or damage, aggressive conduct, and serious violations of rules, constitute direct causes of placement in the CWS. In addition, several adverse experiences (eg, multiple placements and maltreatment) during the time of placement may also contribute to the worsening of externalized disorders that are already present or the promotion of the emergence of such disorders. These results elicit some concerns for the children and adolescents in the CWS regarding the poor prognoses for these disorders that including snow-balling negative outcomes, such as the risk of developing antisocial personality disorder and substance use disorders.^15,46–48^ These disorders are known to be risk factors for delinquency, interactions with the juvenile justice systems, and homelessness.^49^ For example, in France 25% of homeless people and 20% of adults in jail were formerly youths in the CWS.^50,51^

The prevalence of internalized disorders was far from uncommon; 18% of the subjects had anxiety disorder, and 11% had depressive disorder, and these percentages are approximately 3- and 4-fold greater than those of the general population, respectively.^2^ Therefore, it is necessary to acknowledge the gravity of these health problems in this population, particularly considering deleterious effects of these problems on psychosocial functioning and quality of life and their associations with increased suicide rates and drug- and alcohol-use disorders.^52,53^

The high prevalence of externalized and internalized disorders is in line with studies that have reported that attachment insecurities nonspecifically contribute to many types of psychopathologies.^41,54^ Our findings highlight the complexity of screening and care in this population in which externalized and internalized disorders are associated and complexly entangled.

Our meta-analysis identified significant heterogeneity across all of the reported random effect models. Comparable levels of heterogeneity have been identified in other recent systematic reviews and meta-analyses in the general population.^2,55,56^ The significant heterogeneity was attributable to several factors, including country, sex ratio, mean age, sample size, informants, diagnostic criteria, and functional impairment. The rate of any mental disorder was higher in Europe than in the US. Culture may influence the identification and interpretation of symptoms and their attributed meaning.^2^ However, we cannot exclude the possibility that this heterogeneity resulted from structural and organizational differences between Europe and the United States of America. Indeed, alternatives to institutional care, such as kinship care, have been developed in the United States of America but not in some of the European countries included in our meta-analysis,^5,57,58^ and it was not possible to adjust for the structure of placement in our multiple meta-regressions. The prevalence of any mental disorder was higher among the males, but the sex ratios were not significantly different for the individual diagnoses. This finding contradicts the literature regarding the general population, which indicates that there do not appear to be sex differences in the overall prevalence of mental disorders, but there are significant differences in the patterns and symptoms of the disorders (ie, higher prevalences of internalized disorders among girls and externalized disorders among boys).^59^ The absence of differences between girls and boys suggests that externalized disorders in girls and internalized disorders in boys deserve increased attention by professionals and may be underdiagnosed and undertreated.

The other factors related to the heterogeneity (ie, sample size, informants, diagnostic criteria, and functional impairments) may contribute to the methodological issues that future epidemiological studies should consider to produce more accurate estimates. The sampling strategy, including the sample size, is a major factor in the generalization of the estimated prevalence to the entire population. In our work, greater sample sizes were associated with lower estimates of anxiety disorders, which suggests that the estimates may have been overvalued in several studies with small samples. We observed lower estimates of the prevalence of major depressive disorder when the informants were parents, caregivers, teachers, children or adolescents compared with children and adolescents alone. This finding is not surprising; the concordance between informants is known to be low,^2^ and children and adolescents generally report more internalizing disorders than parents, who tend to report more externalizing disorders.^60^ The challenge is thus to provide a strategy for reliable diagnoses that integrate information from different sources. Although diagnostic criteria were standardized and this was one of criteria for selecting the articles, we observed that differences in the diagnostic criteria resulted in differences in the prevalence rates as previously reported in several meta-analyses.^2,61,62^ In a recent study using 2 major nosological systems, the DSM-IV-TR consistently classified more children and adolescents than ICD-10 with an anxiety disorder.^63^ Lastly, functional impairment measurements result in differences in prevalence rates that include lower prevalence estimates for any depressive disorder. Previous studies have reported that the inclusion of an impairment criterion has a significant influence on reducing the prevalence rates of mental disorders, particularly for internalizing disorders.^64^ Surprisingly, we observed the opposite effect of higher prevalence estimates for ODD. However, this association was moderate in strength. The presence of both symptomatic and impairment criteria appears to be the most robust approach for case definition.^65^

Limits. We observed a relatively low number of psychiatric epidemiological surveys that employed standardized diagnostic criteria and psychiatric interviews (n = 8). In contrast, 2 recent reviews identified 41 surveys of the general population that explored the prevalence of mental disorders in children and adolescent over 27 countries^2^ and 174 surveys of adults over 63 countries.^56^ In addition, the methodological quality of the included studies was heterogeneous, and few studies used a whole population approach with random selection. Most of these studies were from single towns or regions and focused on out-of-home children and adolescents, which thus limited the generalizability of the findings. The exploration of heterogeneity was limited by the relatively low number of studies and the lack of information about covariates in several of the studies. Only 5 studies presented the mean age, and only 6 presented the sex ratio, despite the importance of these data. Several important covariates had too many categories and could not be included in the meta-regression analyses (eg, the type of placement, the diagnostic instrument, the type of functional measurement). Other data were not available (eg, age of first placement, the number of changes in placement, and adverse childhood experiences such as maltreatment and serious neglect). These characteristics that we were unable to test might be responsible for the heterogeneity and should be accounted for in future studies because some previous research has reported the importance of maltreatment and adverse experiences in the development of mental disorders.^12,66^

A potential source of bias was the implementation of appropriate search strategies to identify the relevant studies. Specifically, there are important variabilities in the organizational and denomination structures in the child welfare systems between countries that make searches and comparisons difficult. Concordantly, there was evidence of moderate publication bias based on the inspections of the funnel plots, although these results were found to be statistically nonsignificant. Lastly, the majority of the studies did not report prevalence estimates for less-frequent mental disorders, such as eating, elimination, obsessive-compulsive, psychotic, and substance-use disorders. For example, some of the studies reported noticeable rates of psychotic symptoms and highlighted the necessity of reporting them in future research and improving their early detection.^10,11^ The reports of comorbid disorders were also inconsistent between studies, and the high rates of the associations of multiple mental disorders in the studies that reported such rates highlight the fact that these association should be investigated in more detail in future works.^10,15^

Perspectives. The availability of accurate epidemiological data about children and adolescents in the CWS appears to be necessary to guide public policy. Interestingly, the “Best Practices for Mental Health in Child Welfare Consensus Conference” published in 2009 developed guidelines in 5 key areas including systematic screening and assessment.^67^ This systematic screening and assessment could serve as basis for the creation of national registries that could enable more accurate tracking. Altogether, these findings highlight the need for additional studies that specifically target children and adolescents in the CWS to improve the diagnoses and treatments of mental disorders in this population.

## CONCLUSION

Although the high prevalences that were reported for mental disorders in children and adolescents involved in the CWS highlight the need for qualified service provisions, the substantial heterogeneity of our findings is also indicative of the need for accurate epidemiological data to inform and guide effective public policy. Given the importance of mental disorders in this particular population, the poor prognoses of the relevant complex mental states and the high cost to society, it is unfortunate that this population of youths suffering from mental disorders in the CWS does not benefit from greater attention. Thus, this population should be investigated in greater detail in future studies.

## Supplementary Material

Supplemental Digital Content
