# Supplementary material for: The Prevalence of Mental Disorders Among Children and Adolescents in the Child Welfare System: A Systematic Review and Meta-Analysis
Source: Medicine (Baltimore). 2016 Feb 18;95(7):e2622. doi: 10.1097/MD.0000000000002622 (PMC4998603; doi:10.1097/MD.0000000000002622)
Supplement: Supplemental Digital Content [file medi-95-e2622-s001.doc]

Appendix - FUNNEL PLOTS

Figure 1. Funnel plot of studies with prevalence for any depressive disorder in Children and Adolescents in the Child Welfare System (CWS).


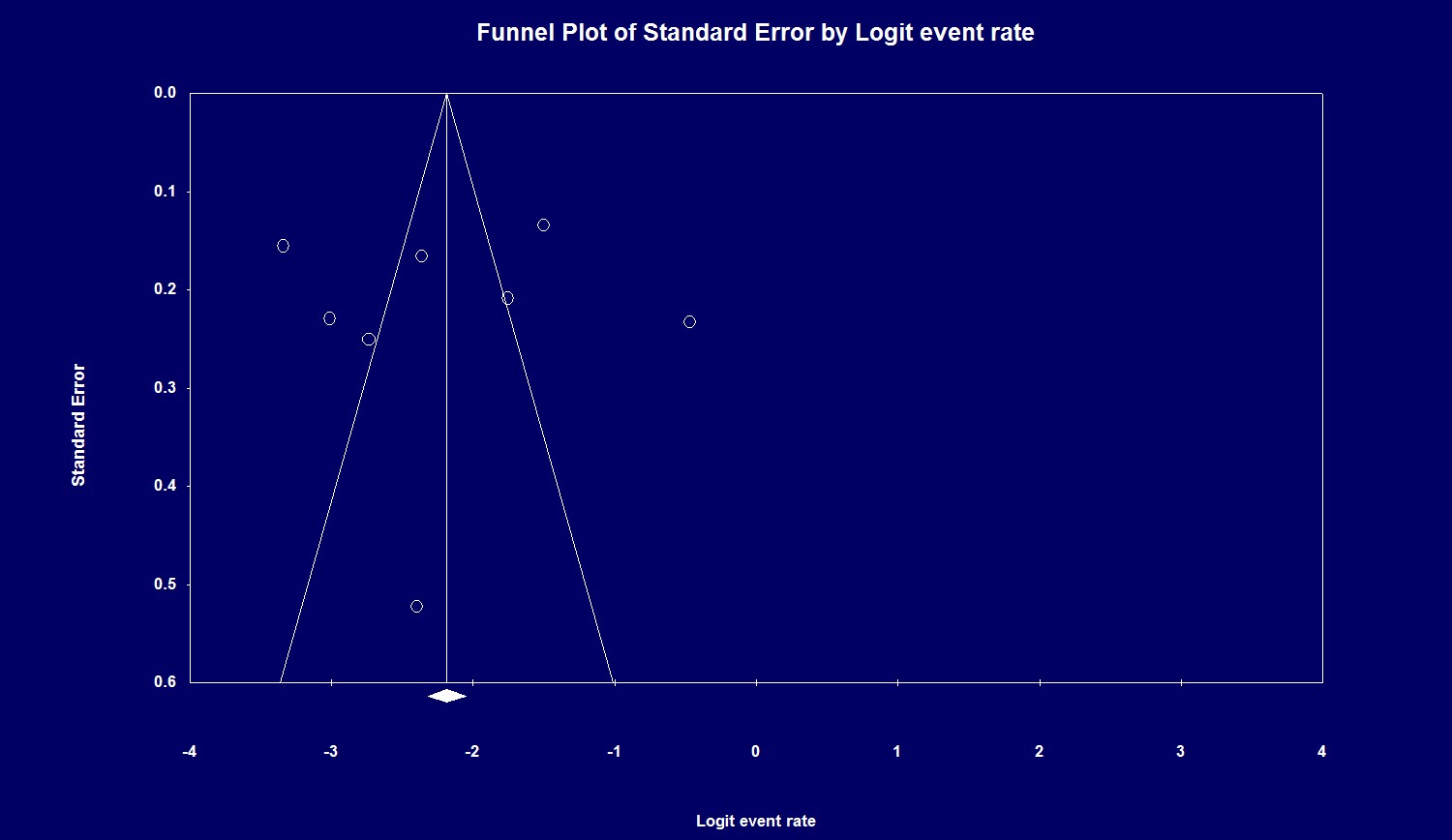


Egger’s regression intercept p=0.97

Figure 2. Funnel plot of studies with prevalence for major depressive disorder in Children and Adolescents in the Child Welfare System (CWS).


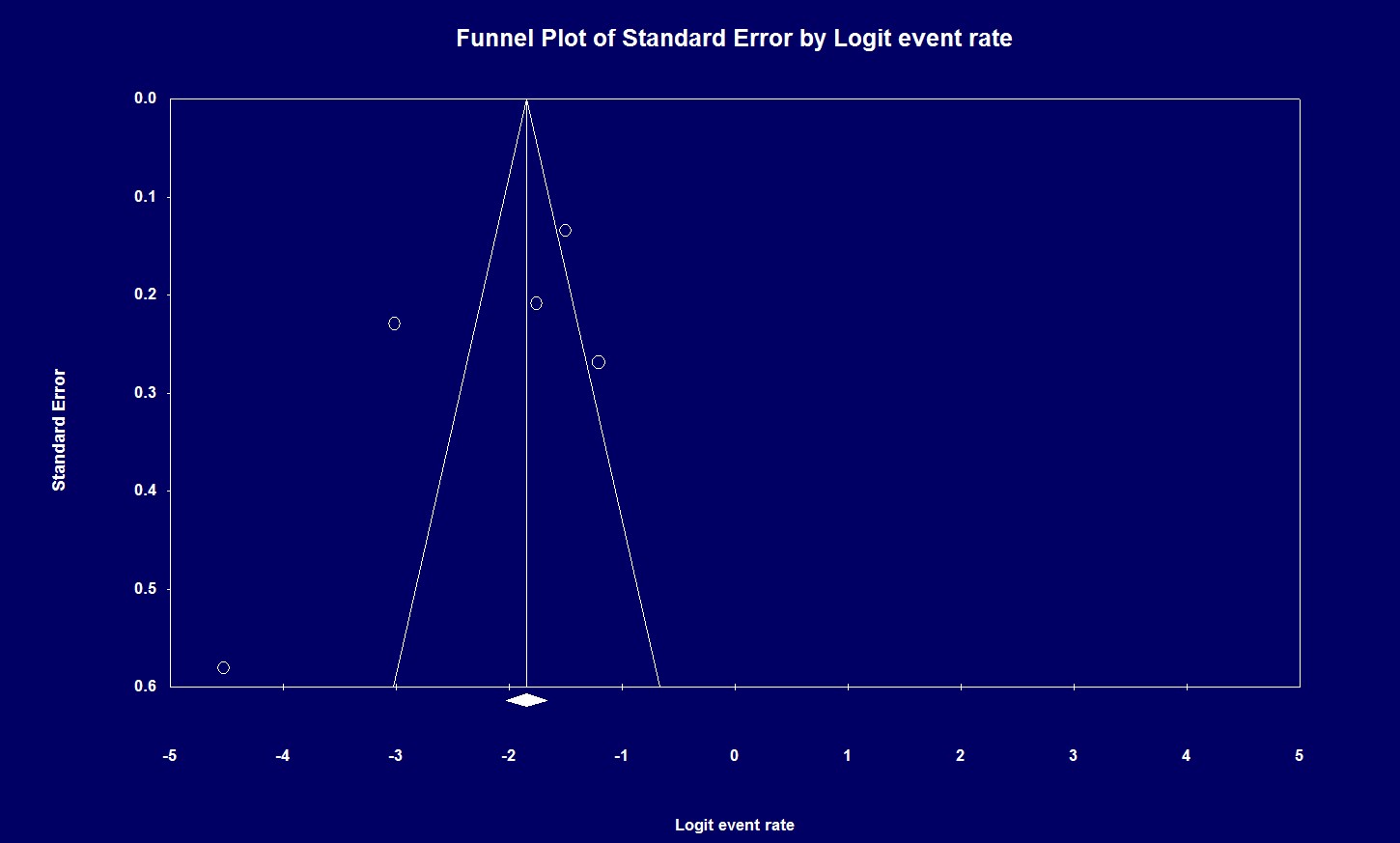


Egger’s regression intercept p=0.24

Figure 3. Funnel plot of studies with prevalence for any disruptive disorder in Children and Adolescents in the Child Welfare System (CWS).


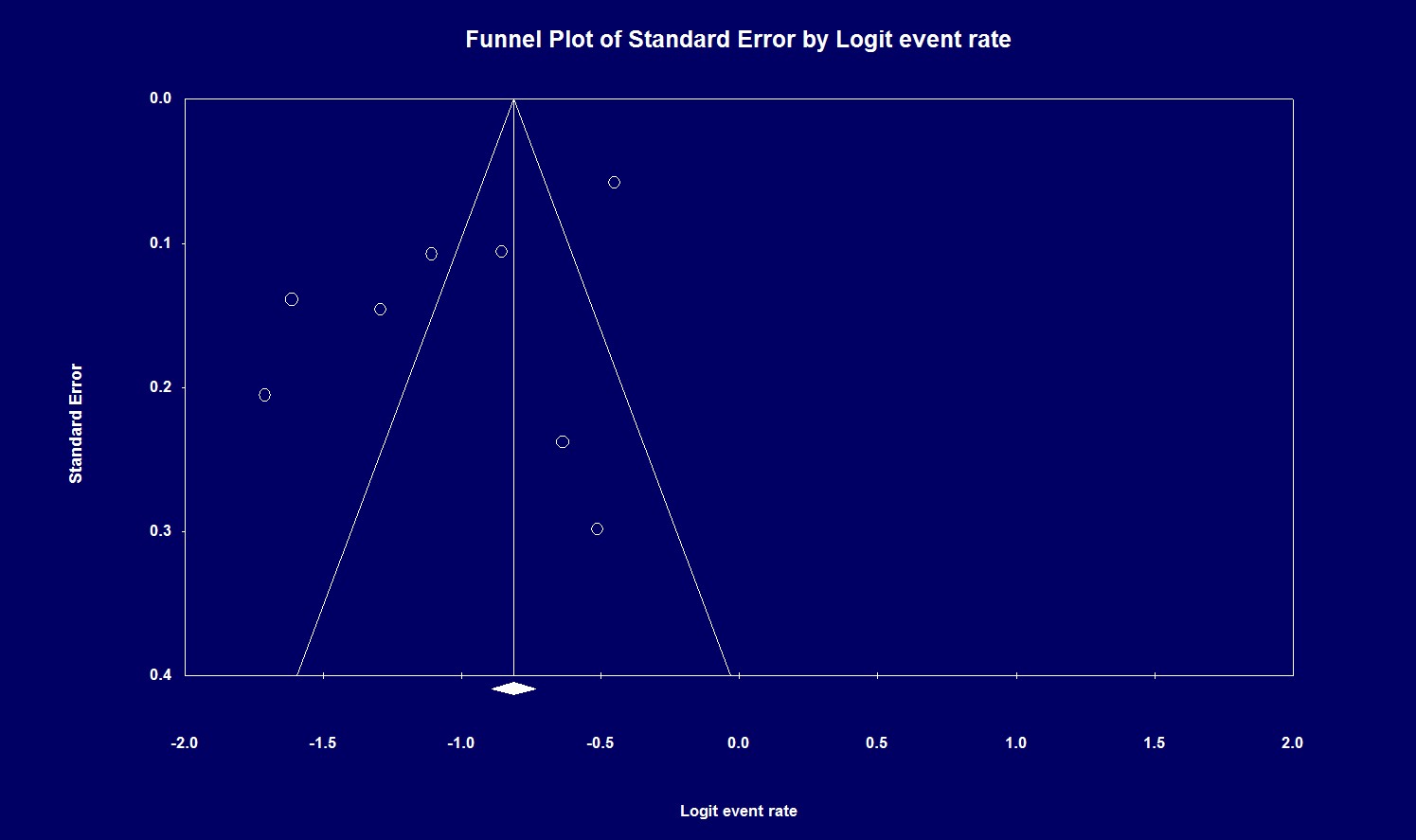


Egger’s regression intercept p=0.11

Figure 4. Funnel plot of studies with prevalence for conduct disorder in Children and Adolescents in the Child Welfare System (CWS).


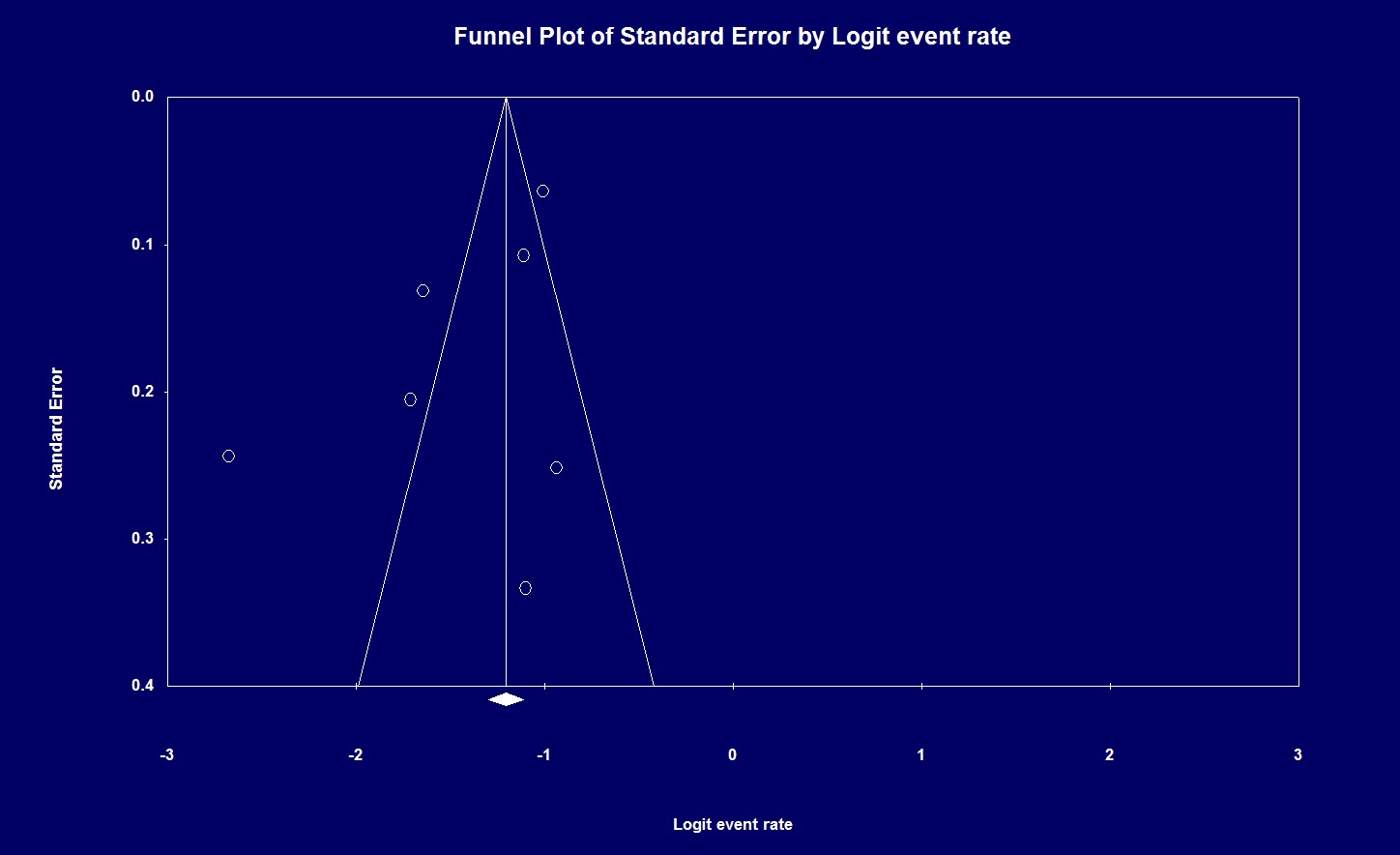


Egger’s regression intercept p=0.16

Figure 5. Funnel plot of studies with prevalence for oppositional-defiant disorder in Children and Adolescents in the Child Welfare System (CWS).


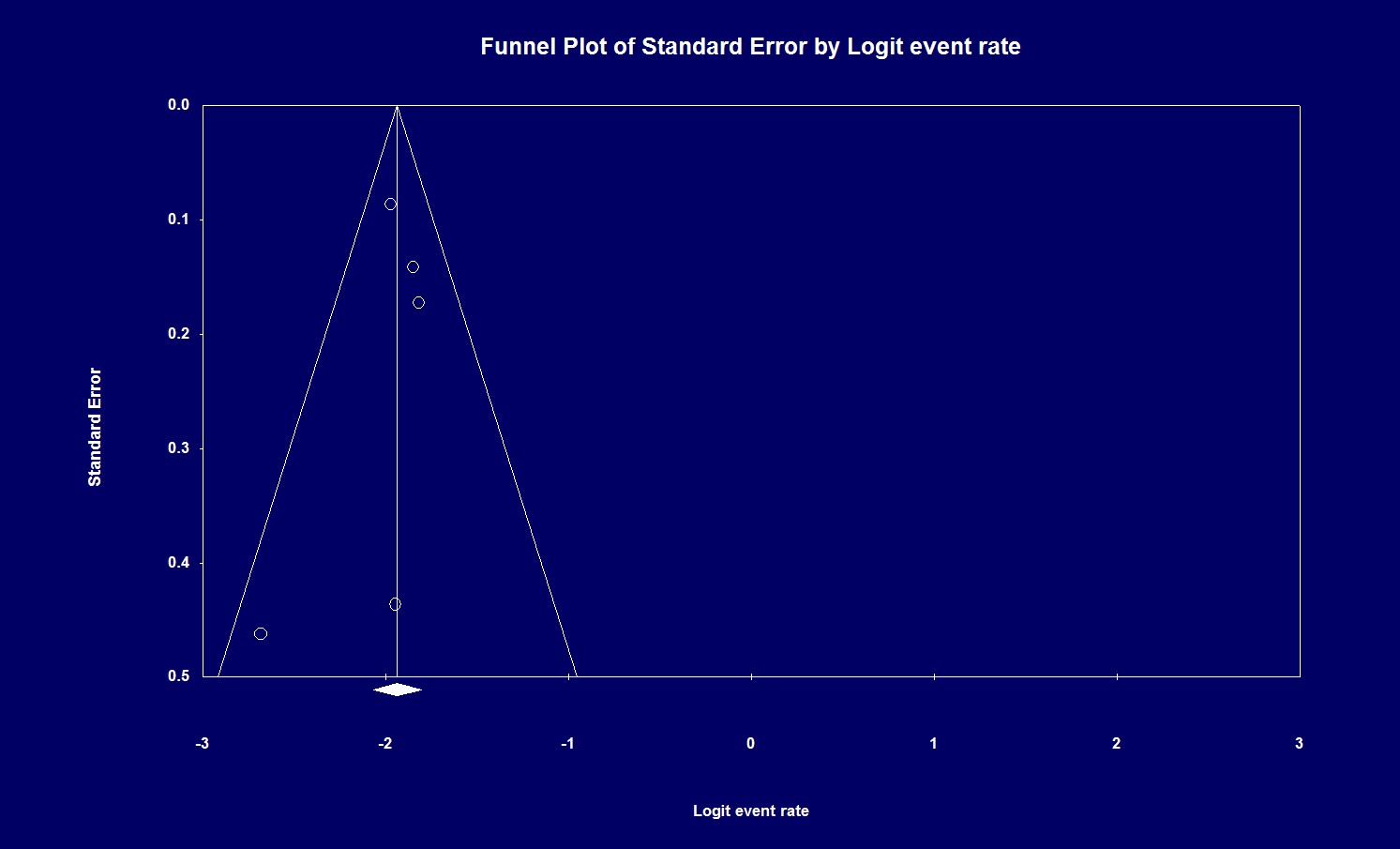


Egger’s regression intercept p=0.58

Figure 6. Funnel plot of studies with prevalence for attention-deficit hyperactivity disorder in Children and Adolescents in the Child Welfare System (CWS).


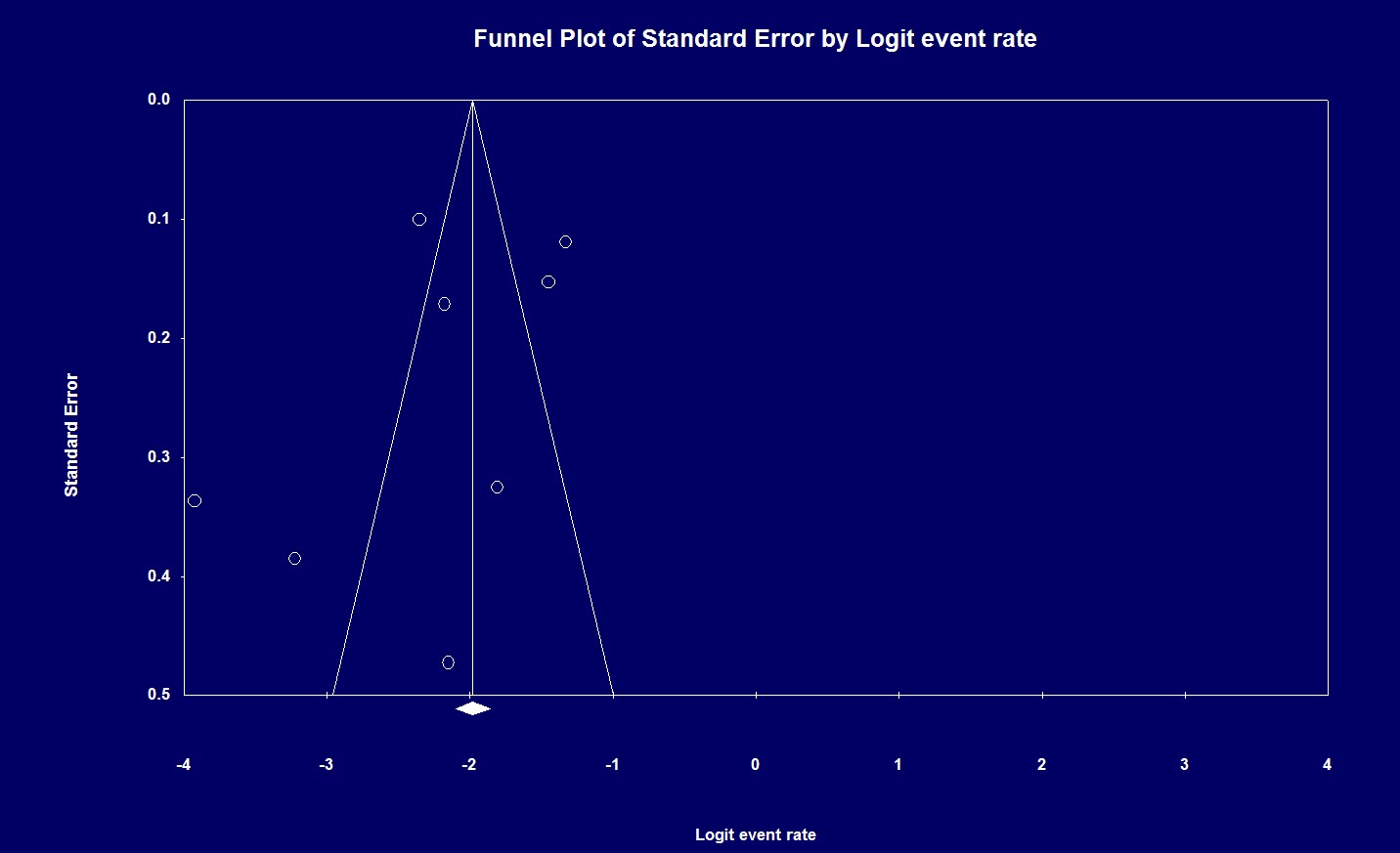


Egger’s regression intercept p=0.38

Figure 7. Funnel plot of studies with prevalence for any anxiety disorder in Children and Adolescents in the Child Welfare System (CWS).


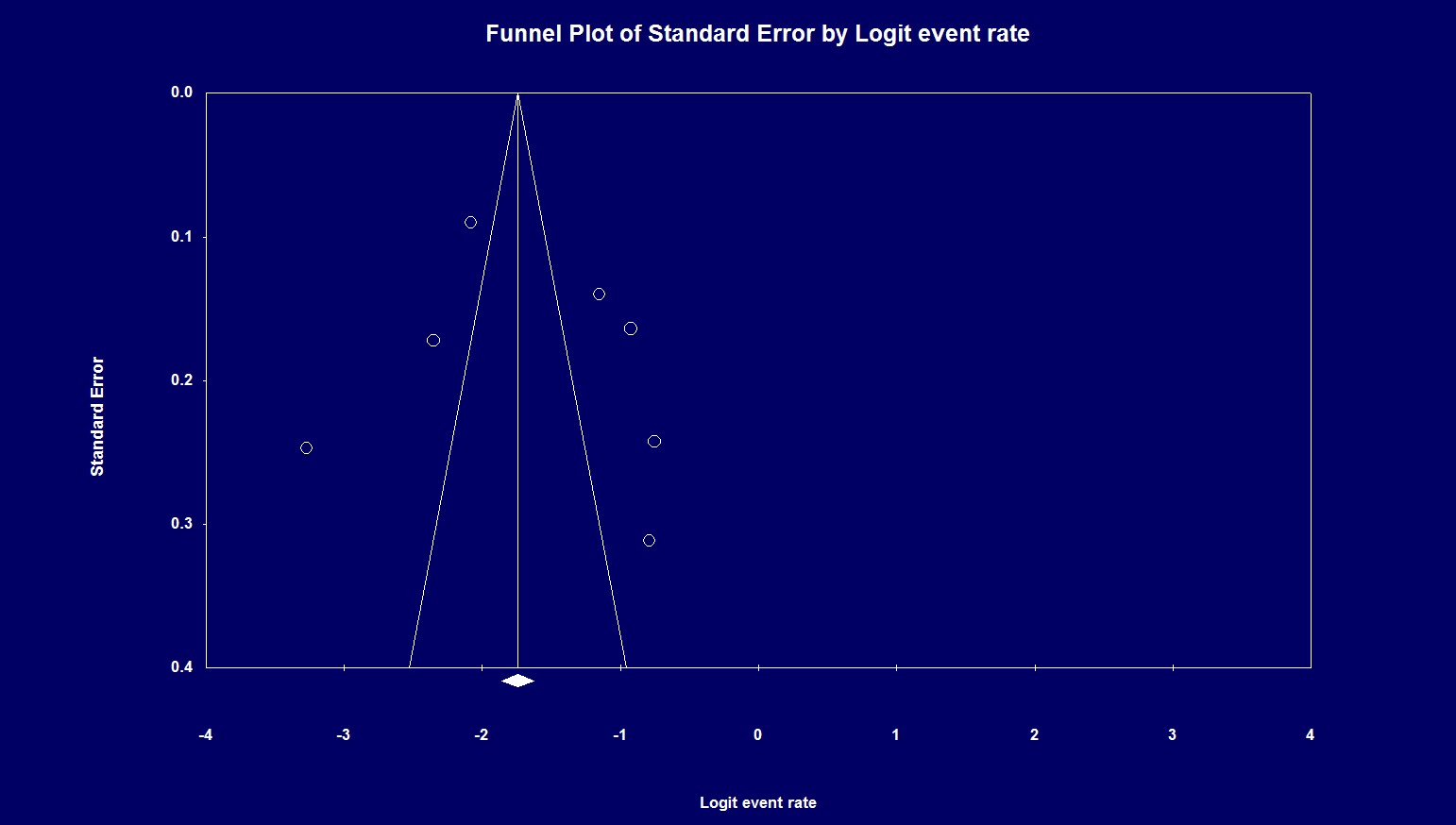


Egger’s regression intercept p=0.59

Figure 8. Funnel plot of studies with prevalence for p[osttraumatic stress disorder](http://en.wikipedia.org/wiki/Posttraumatic_stress_disorder)  in Children and Adolescents in the Child Welfare System (CWS).


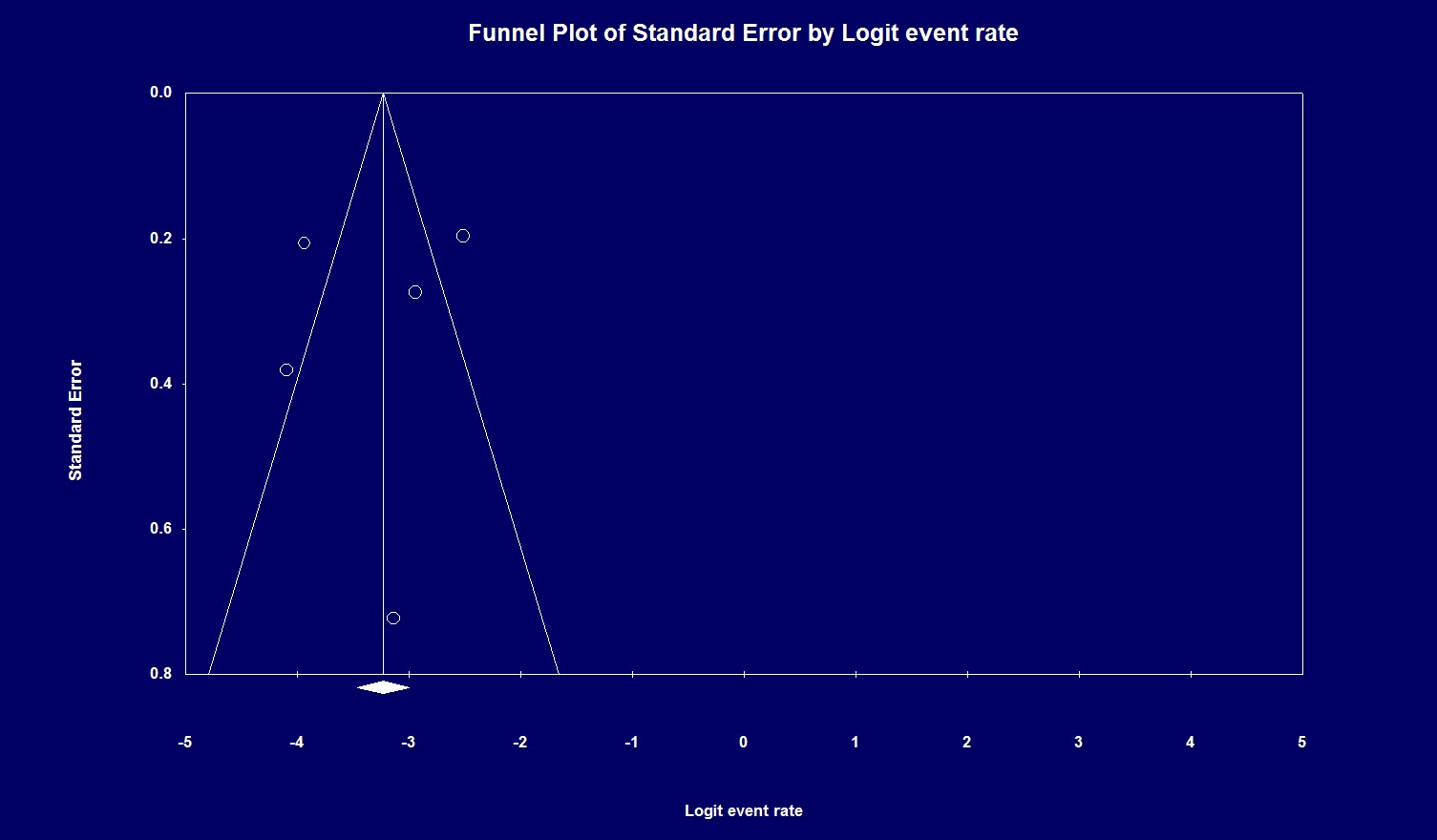


Egger’s regression intercept p=0.76

Figure 9. Funnel plot of studies with prevalence for a[ny disorder](http://en.wikipedia.org/wiki/Posttraumatic_stress_disorder) in Children and Adolescents in the Child Welfare System (CWS).


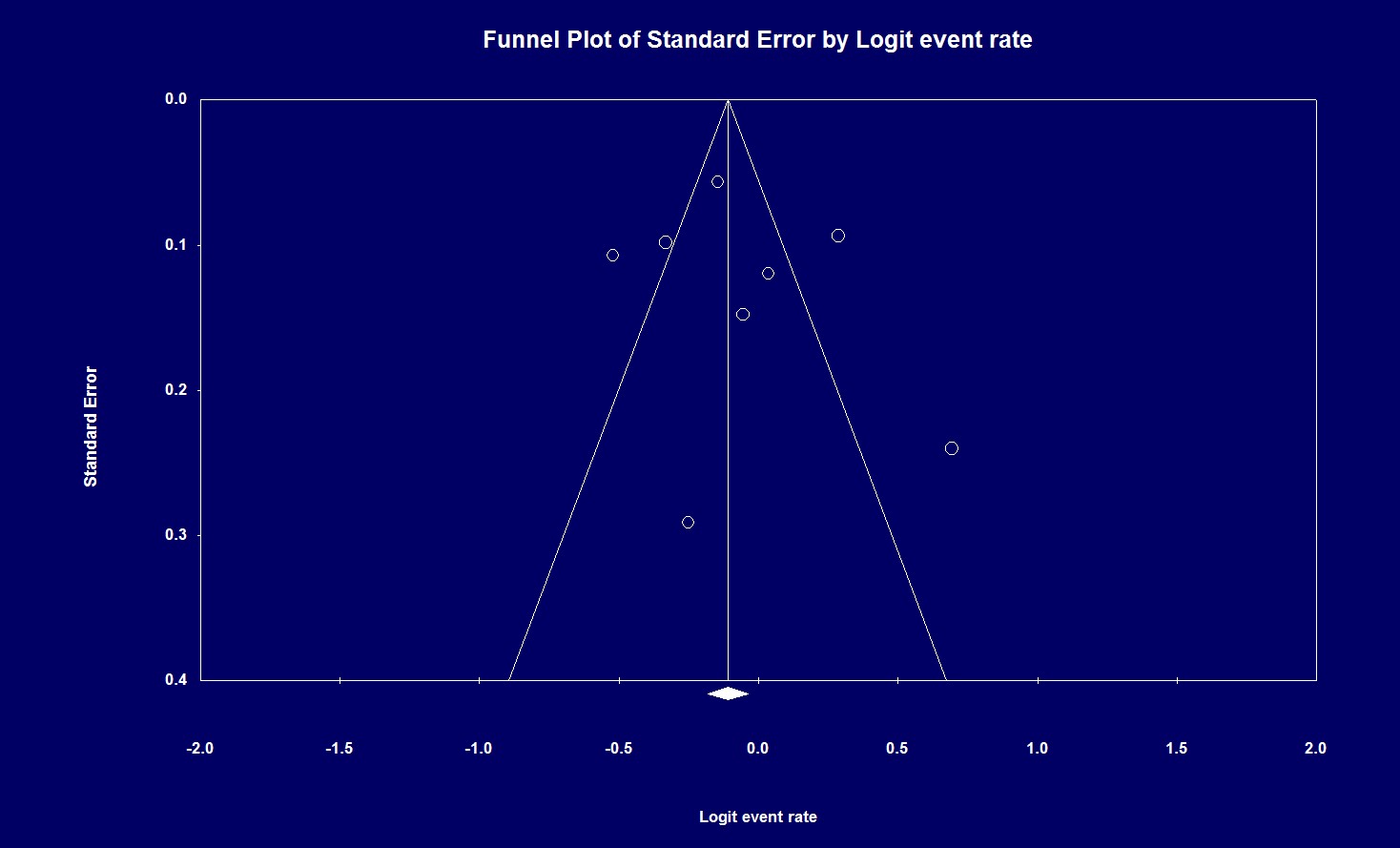


Egger’s regression intercept p=0.59
